# What can we learn from common variants associated with unexpected phenotypes in rare genetic diseases?

**DOI:** 10.1186/s13023-021-01684-w

**Published:** 2021-01-21

**Authors:** Jeanette Erdmann

**Affiliations:** Institute for Cardiogenetics, University of Lübeck, Ratzeburger Allee 160, Building 67, 23562 Lübeck, Germany

**Keywords:** Collagen VI congenital muscular dystrophy, Col VI-CMD, PheWAS, Late-onset risk, Unexpected phenotypes

## Abstract

The purpose of this article is to stimulate discussion about whether a phenome-wide association study is a suitable tool for uncovering late-onset risks in patients with monogenic disorders that are not yet fully recognized because the life expectancy of people with such conditions has only recently extended, and they now reach older ages when they may develop additional complications.

I am well aware that the following analysis has weaknesses and that the results should not be regarded as a definite statement about the late-onset risk for diverticular disease in Col VI-CMD.

My interest is based on having, after almost 45 years without knowing what is causing my slow but ongoing progressive neuromuscular condition, diagnosed myself as a carrier of a pathogenic variant in the *COL6A2* gene, leading to collagen VI congenital muscular dystrophy (Col VI-CMD), using next-generation sequencing and modern information technology [1].

Col VI-CMD is primarily caused by variants in three collagen VI genes, *COL6A1*, *COL6A2*, and *COL6A3* [2, 3], and much less frequently by variants in *COL12A1* [4].

The focus of the clinical course of patients with Col VI-CMD is mostly on the primary pathological phenotype of (slow) progressive muscle weakness, contractures, and hyperflexibility, and respiratory impairment due to exhausted respiratory muscles [5]; however, since collagen VI functions as part of the extracellular matrix [5], it has long been suspected that there are also late-onset disease risks, beyond progressive muscle weakness, such as a higher risk of aneurysms. Also, impairments of the cardiovascular system and intestinal tract are not excluded (Prof. Dr. med. Carsten Bönnemann, personal communication). The functions of collagen VI, so important in muscle disease, may also have implications for obesity, metabolic disease, and cancer in patients with Col VI-CMD (see [6, 7] for detailed reviews) (Fig. 1); however, this has yet to be systematically investigated, as there is currently no sufficiently comprehensive longitudinal registry for patients with this condition. Nevertheless, some unexpected phenotypes caused by rare genetic variants in *COL6A2* and *COL6A3* have been discovered in recent studies; for example, *COL6A2* defects in patients with myoclonus epilepsy [8] and *COL6A3* defects causing dystonia [9].

**Fig. 1:**
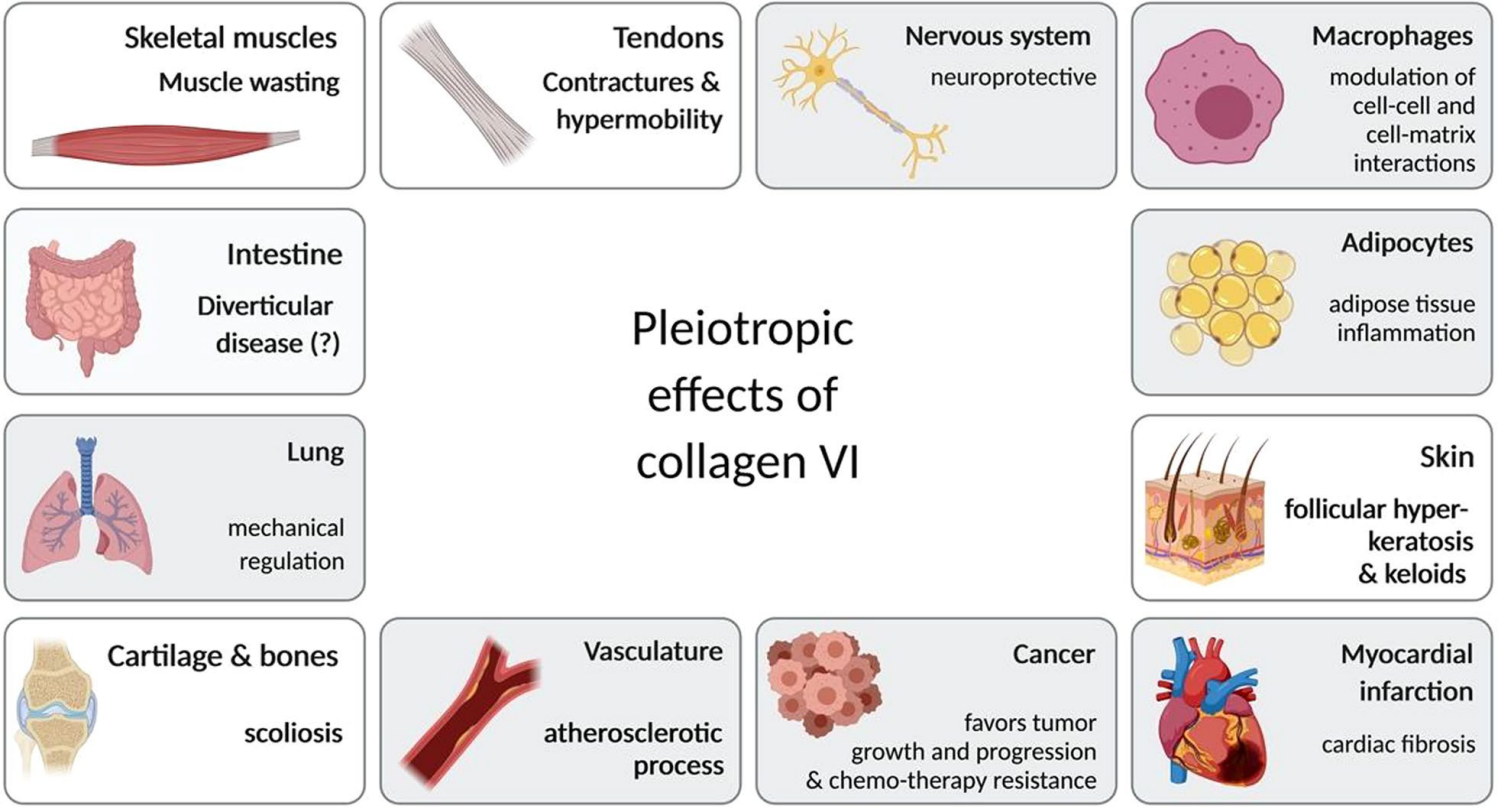
Pleiotropic action of collagen VI in the human body. The tissues and the most prominent phenotypes which constitute COL VI-CMD, based on current knowledge, are skeletal muscle (muscle wasting), tendons (contractures and hypermobility), skin (follicular hyperkeratosis and keloids), and cartilage & bones (scoliosis). However, collagen VI is widely expressed in the human body and may also have important roles in cardiovascular and gastrointestinal disease, obesity, metabolic disease, and cancer. Created with BioRender.com

In general, patients with neuromuscular disorders have a significantly longer life expectancy today than they did a few decades ago, due to better care [10]. Hence, congenital neuromuscular diseases, such as Col VI-CMD or Duchenne muscular dystrophy [11], should now also be considered diseases of adulthood. Consequently, more public health interventions are needed to support such patients and their families as they pass from childhood into adult life. Hence, the early detection of late-onset disease risks, beyond the primary muscle disease, can be vital.

I am well aware of critical health issues that could be related to my condition; 10 years ago, I was severely ill, suffering from acute diverticulitis, a condition characterized by inflammation of one or more diverticula (bulges in the colon wall). In mild cases, diverticulitis can be cured with antibiotics, while in severe cases, surgery is the only therapeutic option. In my case, despite presenting with severe rectal bleeding, leading to fainting and repeated bouts of diverticulitis, my doctors decided not to consider surgery, rather treating me with high doses of antibiotics. This informed decision was made because of general caution regarding anesthesia in patients with neuromuscular disease, and my specific condition, which had required night-time non-invasive ventilation for almost 15 years, due to impaired lung function because of a severely exhausted diaphragm. Since we have decided against surgery, the problem of the diverticula is not really treated, but has hovered over me, like the sword of Damocles, for the last decade, and will continue to do so for years to come.

In 2010, Denny and colleagues suggested the concept of phenome-wide association studies (PheWAS) by performing a “reverse genome wide association study (GWAS)”, thereby determining, for a given genotype, the range of associated clinical phenotypes [12]. This reverse genetic approach can provide novel insights not readily attainable by forward genetic strategies. PheWAS takes advantage of increasingly large sets of human genetic variation data, coupled with dense phenotypic information, to analyze genotype–phenotype associations [13]. In this way, it is possible to generate an almost complete picture of the pleiotropic effects of genetic variations and respective genes, where pleiotropy describes the phenomenon in which a gene influences two or more, seemingly unrelated, phenotypic traits [14]. Before PheWAS was conceptualized, pleiotropy was established through intensive phenotyping of relatively small disease cohorts and, most importantly, by functional studies in mice and human cell culture models. As just one example, genetic variants in *GJA1*, which encodes connexin 43, cause oculodentodigital dysplasia (OMIM #164200), a rare condition characterized by a typical facial appearance and highly variable findings related to the eyes, teeth, and fingers [15].

Within the last decade, several large-scale biobanks have been established worldwide, often with genomic as well as comprehensive phenotypic data, with total enrollment in the largest biobanks surpassing 500,000 individuals [16]. A prime example of genotypic and phenotypic data made publicly available is the UK Biobank (UKBB). UKBB aims to improve the prevention, diagnosis, and treatment of a variety of serious and life-threatening diseases, including cancer, heart disease, stroke, diabetes, arthritis, osteoporosis, eye disease, depression, and dementia [17]. It tracks the health and well-being of 500,000 volunteers and provides health and genetic information to researchers from science and industry. This makes the UKBB the most comprehensive clinical and genetic data resource currently publicly available. Linking the PheWAS approach and UKBB data allows researchers to associate every single genetic variant with more than 3,000 phenotypes stored in the UKBB for each patient. UKBB data can be accessed through several platforms, including http://pheweb.sph.umich.edu/.

Along these lines, two interesting studies have been published very recently, both using PheWAS and data from large biobanks in the context of Mendelian diseases. First, Tcheandjieu and colleagues reported that the spectrum of associations of common and rare variants in genes involved in Mendelian diseases can be extended to individual phenotypes within the general population [18]. This study was based on four well-described syndromic diseases (Alagille, Marfan, DiGeorge, and Noonan syndromes) and PheWAS analysis of UKBB data, and show that specific phenotypes associated with these rare disease genes can also be identified in population-based data by PheWAS.

Even more interestingly, Park et al. [19] used a cohort of > 11,000 unselected individuals from the Penn Medicine Biobank to identify associations of rare variants in the *LMNA* (Lamin A/C) gene with diverse phenotypes using a PheWAS approach. The authors demonstrated that pathogenic *LMNA* variants are an underdiagnosed cause of cardiomyopathy. Intriguingly, they also detected an unreported association between loss of function variants in *LMNA* and renal disease, a phenotype apparently unconnected with cardiomyopathy.

A very convenient way to access UKBB data, in addition to publicly available curated GWAS information, is at https://atlas.ctglab.nl/PheWAS [20]. This website hosts a comprehensive database of publicly available GWAS summary statistics and results from GWAS of 600 traits from UK Biobank release 2. Here, users are able to both access original summary statistics and obtain a variety of results from pre-performed analyses, such as risk loci information, LD regression score [21], MAGMA [22], and multi GWAS comparisons [20].

Leveraging this rich data resource, I performed an exploratory gene-based PheWAS for *COL6A2*, with the aim of identifying potential late-onset risks in patients with Col VI-CMD. My hypothesis is that the association of common genetic variants in *COL6A2* with phenotypes deposited in publicly available GWAS datasets may reveal late-onset disease risks, which could inform future disease management. The results of the PheWAS for *COL6A2* over a broad range of phenotypes are presented in Fig. 2a, b.

**Fig. 2:**
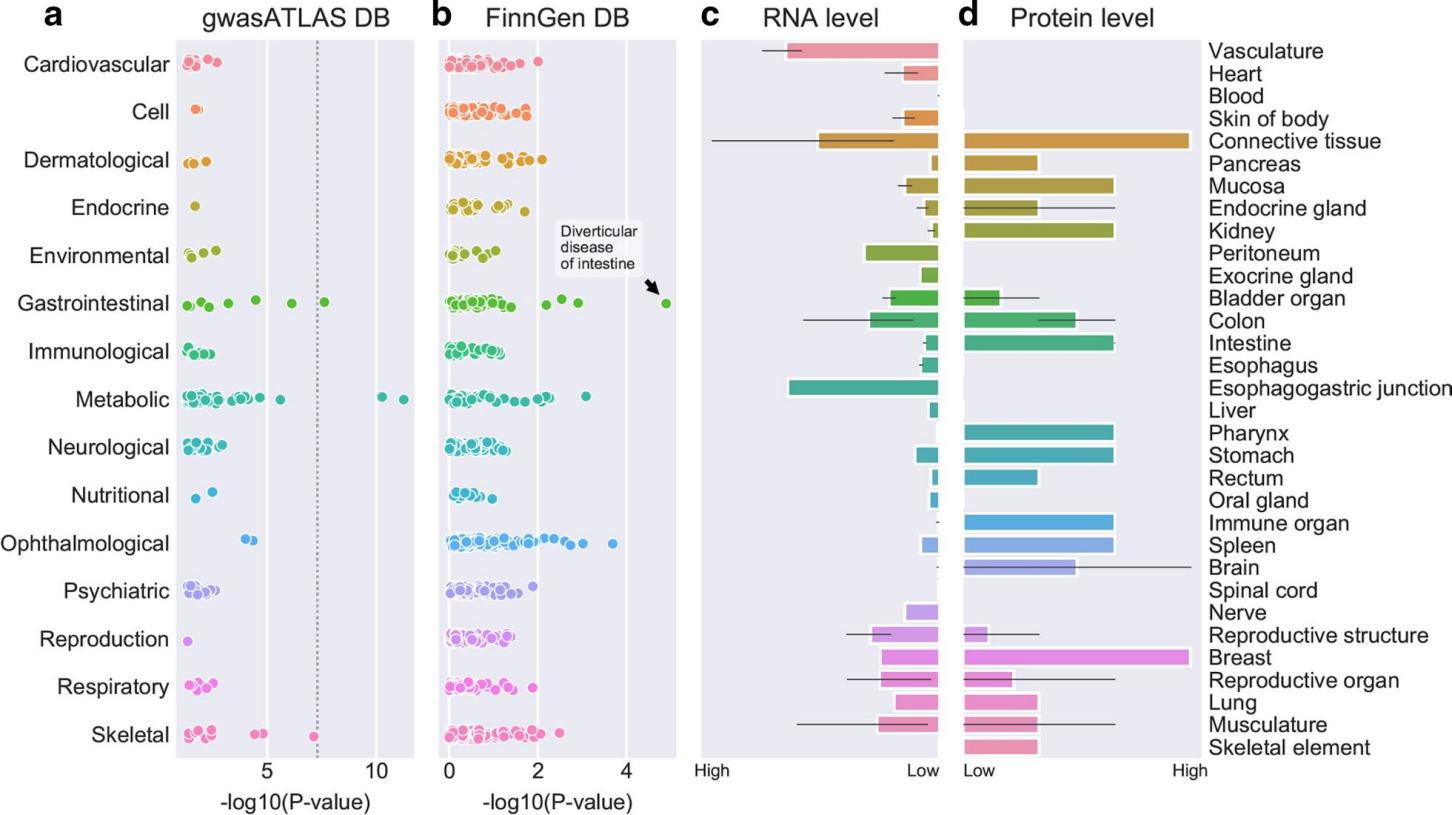
**a**–**d** Results of screening for genetic associations between common variants tagging the *COL6A2* gene and a broad spectrum of phenotypes (**a**, **b**), as well as RNA (**c**) and protein (**d**) expression of collagen type VI, alpha 2. **a** Results of a *COL6A2*gene-based PheWAS. Plot showing association results for rs12626197, a common intronic variant tagging the *COL6A2* gene on chromosome 21, across all phenotypes in the gene atlas database (accessed July 2020). Phenotypes are clustered according to related diseases (e.g., cardiovascular diseases or gastrointestinal diseases). **b** Replication in the FinnGen study. Plot showing the association results for rs12626197 across all phenotypes (clustered as in panel **a**) in the FinnGen database (accessed July 2020). rs12626197 shows an association signal for diverticular disease, thereby replicating the finding from the gene atlas database shown in panel **a**. **c** RNA expression of *COL6A2*. Summary of *COL6A2* RNA expression in normal human tissue based on RNA-seq expression and data from the expression atlas (https://www.targetvalidation.org/target/ENSG00000142173, accessed November 2020). *COL6A2* is expressed in the colon and intestine, as well as other human tissues. **d** Protein expression of collagen type VI, alpha 2. Summary of protein expression in normal human tissue based on the human protein atlas (http://www.proteinatlas.org/ENSG00000142173-COL6A2/tissue, accessed November 2020). Collagen type VI, alpha 2 is expressed in the colon and intestine, as well as other human tissues

The most significant finding is an association between the *COL6A2* gene and waist-hip ratio (*p* = 5.0e−09) [23]. Interestingly, the second most significant genome wide hit was with diverticular disease (*p* = 2.4e−8) [24] (Fig. 2a). Moreover, the association between rs12626197 and diverticular disease could be replicated using data from the FinnGen study (data freeze 3, spring 2019), consisting of 135,638 individuals (accessed November 2020 at http://r3.finngen.fi/) (Fig. 2b).

The association of common variants at the *COL6A2* gene locus with diverticular disease was further supported by publicly available gene and protein expression data. COL6A2 is highly expressed in connective tissue and vasculature at both the RNA and protein levels, but also in colon and intestine (Fig. 2c, d).

To validate these findings, comprehensive patient registries, with a specific focus on secondary (late-onset) phenotypes, are required; however, in the absence of such registries, the link between COL6-CMD and the gut could be studied using animal models, for example, knockouts of *Col6a2* in zebrafish or mice.

In summary, this exploratory PheWAS appears to support the hypothesis that diverticular disease may be a late-onset risk for patients carrying *COL6A2* mutations leading to Col VI-CMD. However, association does not definitively establish a causal relationship between diverticulitis and genetic defects in *COL6A2*, since other genetic and environmental factors (e.g., reduced activity levels, diet, etc.) may contribute.

It is my intention to stimulate systematic studies of whether late-onset risks in monogenic disorders can be uncovered by PheWAS analysis.

## Data Availability

Not applicable.

## Abbreviations

Col VI-CMD: Collagen VI congenital muscular dystrophy
GWAS: Genome wide association study
PheWAS: Phenome-wide association study
UKBB: UK Biobank
